# Resource Reallocation of Two Grass Species During Regrowth After Defoliation

**DOI:** 10.3389/fpls.2018.01767

**Published:** 2018-12-05

**Authors:** Yanshu Liu, Xiaohui Yang, Dashuan Tian, Richun Cong, Xiao Zhang, Qingmin Pan, Zhongjie Shi

**Affiliations:** ^1^Institute of Desertification Studies, Chinese Academy of Forestry, Beijing, China; ^2^Institute of Geographical Sciences and Natural Resources Research, Chinese Academy of Sciences, Beijing, China; ^3^Inner Mongolia Research Center for Prataculture, Chinese Academy of Sciences, Beijing, China

**Keywords:** biomass allocation, grazing, carbohydrates, protein, grassland management

## Abstract

Defoliation is widely used for grassland management. Our understanding of how grass species adjust their regrowth and regain balance after defoliation remains limited. In the present study, we examined the regrowth processes of two dominant species after defoliation in grasslands in Inner Mongolia. Our results showed that the aboveground biomass and total biomass of both species significantly decreased and did not completely recover to the control level after 30 days of regrowth. The leaf mass ratio of *Leymus chinensis* reached the control level at 15 days, but that of *Stipa grandis* did not recover to the control level. The root mass ratio of these species reached the same levels as that of the control plants within 10 days after defoliation. As indicated by the dynamics of water-soluble carbohydrates (WSCs), protein, and biomass-based shoot: root ratios, both species regained balances of WSCs and protein between above- and below-ground organs at day 10 after defoliation; however, the biomass regained balance 15 days after defoliation. We deduced that the biomass-based shoot:root ratio was regulated by the WSCs and protein concentrations. In conclusion, following defoliation, both grass species first restore their nutrient-based balance between above- and below-ground parts and then regain biomass balance.

## Introduction

Defoliation caused by animal grazing and hay production has profound impacts on plant growth and development in grassland ecosystems. In general, defoliation decreases the total leaf area, plant photosynthesis, and uptake and assimilation of nutrients, such as carbon and nitrogen, but increases the mobilization of reserved nutrients to develop new leaves and stems (Macduff and Jackson, 1992; Volenec et al., 1996). Previous studies have suggested that defoliation can stimulate plant regrowth (McNaughton, 1983; Muthoni et al., 2014). In fact, plants have evolved a suite of morphological and physiological mechanisms to cope with defoliation. The ability of plants, which use internal stores of carbon and nitrogen, both to rapidly restore photosynthetically active leaf area and to meet the maintenance demands of other organs, is among the key factors that facilitate plant survival during the first 2 weeks of regrowth after defoliation (Volenec et al., 1996).

Numerous studies have highlighted the importance of water-soluble carbohydrate (WSC) reserves of grass species during regrowth following defoliation (Hume, 1991; Donaghy and Fulkerson, 1997). For some grass species, WSCs in the stem stubble (residual stem after defoliation) constitute the main source for regrowth (Steen and Larsson, 1986); in contrast, other studies have found that WSC reserves in the rhizomes play a critical role in the regrowth of rhizomatous grasses after defoliation (Wang, 2007). For plants subjected to defoliation, WSCs are usually affected by both stubble height (Fulkerson and Slack, 2003; Lee et al., 2009; Jones et al., 2017) and the temporal interval between defoliations (Singh and Sale, 1997; Donaghy and Fulkerson, 2002).

In addition to WSCs, nitrogen reserves also play an important role in plant regrowth (Ourry et al., 1994; Volenec et al., 1996). In herbaceous plants, protein is an important mobile storage form of nitrogen (Staswick, 1994). Thornton et al. (1993) reported that, in support of the growth of new leaves after defoliation, the remobilization of protein reserves and nitrogen uptakes varies among grass species. It has been recognized that the plant root: shoot ratio may function as a balance in terms of resource acquisition and allocation (Agren and Ingestad, 1987). Plants first transport and use their pre-defoliation reserves for maintenance and regrowth within the first 2 weeks after defoliation, after which the newly growing leaves then assimilate new carbohydrates and allocate them to different parts of plants (Detling et al., 1979; Menke and Trlica, 1981; Detling and Painter, 1983). However, WSCs and proteins, during regrowth following defoliation, are often examined separately (Augustine et al., 2011).

Defoliation usually causes a great decline in the plant shoot:root ratio due to the loss of aboveground parts, which disrupts the balance between above- and below-ground parts. To recover from an unbalanced state for biomass loss after defoliation to a plant’s original state, in terms of biomass allocation between above- and below-ground parts, a defoliated plant usually exhibits a higher relative growth rate (RGR) of aboveground organs. Such phenomena have been extensively observed (Meyer, 1998; Zhao et al., 2008); however, the rate at which plant species adjust their biomass allocations between above- and below-ground organs after defoliation remains unclear. Moreover, the role of nutrients, such as WSCs and proteins, in the rebalancing process of biomass allocation between above- and below-ground organs has rarely been addressed.

*Leymus chinensi*s (Trin.) Tzvel. and *Stipa grandis* P. Smirn. are two widely distributed species in Inner Mongolia’s grasslands (Liu et al., 2012). *L. chinensis*, a rhizomatous grass species, has long, strong rhizomes and exhibits vigorous vegetative propagation; thus, this species usually gives rise to extensively spreading clones and often forms monodominant stands in relatively wet habitats. In contrast, *S. grandis*, a typical bunch grass, usually occupies and dominates relatively dry habitats (Briske and Derner, 1998). The belowground parts of these two species often function as organs for nutrient acquisition and storage, in addition to water absorption from the soil, to address frequent drought stresses. Nutrients stored within roots or rhizomes allow plants to easily overcome fluctuations in nutrient availability. Via the phalanx strategy (producing a compact cluster of closely spaced ramets) (Cheplick, 1997; Chen et al., 2011), *S. grandis* can monopolize and consolidate locally available resources, which is beneficial in a competitive environment. Both species are well-adapted to grazing and periodic drought but differ in terms of functional types (rhizomatous grass vs. bunchgrass). The aim of this study was to answer the following questions: (1) How different is the regrowth between two functional types after defoliation? (2) How do these species reallocate their biomass, WSCs, and proteins between their above- and below-ground parts after defoliation? (3) Does biomass rebalancing between above- and below-ground parts keep pace with the rebalancing of WSCs and proteins? This study will provide a theoretical reference to formulate a wiser grazing system in Inner Mongolia’s grasslands.

## Materials and Methods

This experiment was conducted at the Inner Mongolia Grassland Ecosystem Research Station, Chinese Academy of Sciences (IMGERS), in 2014. The station is located within the Xilin River watershed in the Inner Mongolia Autonomous Region, China (116°40′40^′′^ E, 43°32′45^′′^ N, 1250–1280 m a.s.l.). The area has a semiarid continental temperate steppe climate, which consists of a dry spring season and a humid summer. The average annual temperature is 0.92°C and the average annual precipitation is 337 mm; rainfall occurs mostly within the period from June to August.

Seeds of two species were collected from a permanent fenced grassland plot with an area of 25 ha. To break dormancy, the seeds were immersed in low-temperature water for 12 h and then sown in pots (280 mm in diameter and 260 mm in depth) filled with chestnut soil and arranged in an open field on June 1. A total of 100 pots for each species, with a density of 30 plants per pot, were planted and watered every 3 days during the first 2 weeks and then irregularly depending on the soil conditions. On July 5, 20 uniform seedlings were kept in each pot; other seedlings were removed.

On August 10^th^, 30 pots for each species were defoliated to a stubble height of 3 cm; another 30 pots for each species were chosen as the controls. To track the regrowth processes of these two species after defoliation, we harvested all the above- and below-ground parts in three pots for each species at 0, 1, 2, 3, 5, 10, 15, 20, 25, and 30 days after defoliation. The aboveground parts were collected and divided into leaves and stems, while the belowground parts were collected via water washing and meshed by a 1 mm × 1 mm screen; *L. chinensis* was further divided into root and rhizome. All plant materials collected were freeze-dried until at a constant weight and then ground with a ball mill (Retsch MM 400; Retsch, Haan, Germany) for WSC and protein analysis. The protein concentration of each organ was analyzed with a Nitrogen Analyzer System (KJELTEC 2300 AUTO SYSTEM II, Foss Tecator AB, Höganäs, Sweden). The protein concentration at plant level was calculated as the biomass weighted average protein concentration of the leaf, stem, root, and rhizome.

The leaf mass ratio (LMR) was calculated as LMR = (leaf weight)/(total biomass), and the root mass ratio (RMR) as RMR = (root weight)/(total biomass). The RGR in terms of the aboveground biomass was calculated as RGR = (lnB2–lnB1)/(t2–t1), where B1 and B2 are the aboveground biomass measured at time 1 and time 2, respectively.

To determine the WSC concentrations of leaf, stem, root and rhizome for two species, 100 μg of lyophilized plant tissue powder was suspended in 5 ml of distilled water and incubated in 100°C boiling water for 30 min. After the suspensions had cooled to room temperature and been centrifuged (5 min at 10000 *g*), the supernatants were removed and transferred to 50-ml volumetric flasks, after which the pellets were re-extracted one time. The supernatants were subsequently combined in a 50-ml volumetric flask, diluted with water to volume, and mixed (Solution A). Then, 500 μl of Solution A and 1.5 ml of distilled water were added to a new tube, after which 0.5 ml of a throne reagent and concentrated sulfuric acid were added. The contents of the tube were then mixed together, after which the tube was incubated in 100°C boiling water for 1 min. After the tube was cooled to room temperature, the concentration of WSCs was measured by a photoelectric colorimeter (Beckman Coulter DU800, Brea, CA, United States) (Li et al., 2000).

In the current study, we used the shoot:root ratios of the biomass, WSCs, and proteins in the control treatment as references of a balanced state; then, we defined the rebalance of biomass, WSCs, and proteins as the state at which the corresponding shoot:root ratio of a defoliated plant recovered to the level of control treatments, i.e., no significant difference in shoot:root ratio between defoliated treatments and control treatments.

We performed ordinary least squares regressions to examine the relationship between the biomass-based shoot:root ratio with the WSCs concentration, amount of WSCs per plant, protein concentration, and protein amount per plant. The differences between the defoliation and control treatments over regrowth days were measured with repeated measures ANOVA and were compared between two species with a *t*-test. All statistical analyses were performed using SAS version 9.1 (SAS Institute, Cary, NC, United States).

## Results

### Defoliation Impacts on Plant Height and the Number of Tillers per Plant

**FIGURE 1:**
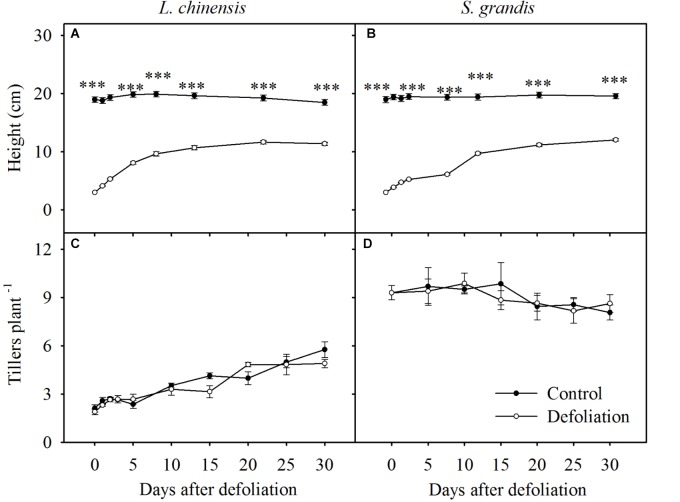
Seedling height and number of tillers per plant of *L. chinensis* **(A,C)** and *S. grandis* **(B,D)** after defoliation. Values are means ± SE (*n* = 3). ns, ^∗^, ^∗∗^, and ^∗∗∗^ indicate non-significant difference at *P* < 0.05, *P* < 0.01, and *P* < 0.001, respectively.

Defoliation significantly decreased the height of *L. chinensis* and *S. grandis* during the 30 days of regrowth (Table 1). The height of *L. chinensis* and *S. grandis* plants in the defoliation treatment increased rapidly and reached half that of the control plants within the first 10 days after defoliation, after which the height gradually plateaued (Figure 1). No significant effects of defoliation on the number of tillers per plant were observed in either species, but the number of tillers increased in *L. chinensis* regardless of defoliation treatment.

**Table 1:** *F*-values and *P*-values of repeated measures ANOVA on the effects of defoliation and days after defoliation on height, number of tiller, LWR, RWR, RGR in terms of aboveground biomass, aboveground biomass, total biomass, biomass-based, WSC-based, and protein-based shoot:root ratios of *L. chinensis* and *S. grandis.*

Variable	Days		Defoliation		Days × Defoliation	
	*F*	*P*	*F*	*P*	*F*	*P*
*L. chinensis*					
Heigh	43.19	<0.0001	3454.24	0.0108	37.72	<0.0002
Number of tiller	30.21	<0.0001	1.45	0.2355	1.6	0.148
LWR	6.17	<0.0001	35	<0.0001	8.74	<0.0001
RWR	18.09	<0.0001	149.45	<0.0001	17.98	<0.0001
Aboveground biomass	53.64	<0.0001	53.01	<0.0001	1.03	0.4258
Total biomass	43.33	<0.0001	50.59	<0.0001	2.89	0.0272
Biomass-based shoot:root ratio	14.09	<0.0001	5.26	0.0295	8.84	<0.0001
WSCs-based shoot:root ratio	12.01	<0.0001	0.53	0.4723	1.67	0.1672
Protein-based shoot:root ratio	7.4	<0.0001	0.62	0.437	5.94	0.0004
*S. grandis*					
Heigh	48.88	<0.0001	4465.21	0.0095	39.6	<0.0002
Number of tiller	1.33	0.2789	0.01	0.9323	0.28	0.9404
LWR	5.36	0.004	17.38	0.0003	0.07	0.9778
RWR	1.13	0.3651	61.68	<0.0001	5.31	<0.0001
Aboveground biomass	11.81	<0.0001	32.15	<0.0001	1.04	0.4218
Total biomass	15.25	<0.0001	27.71	<0.0001	1.51	0.2126
Biomass-based shoot:root ratio	2.65	0.0366	6.28	0.0183	5.46	0.0008
WSCs-based shoot:root ratio	3.77	0.0093	24.21	<0.0001	7.57	0.0001
Protein-based shoot:root ratio	1.38	0.26	5.37	0.0286	7.83	<0.0001

### Defoliation Impacts on Leaf Mass Ratio and Root Mass Ratio

**FIGURE 2:**
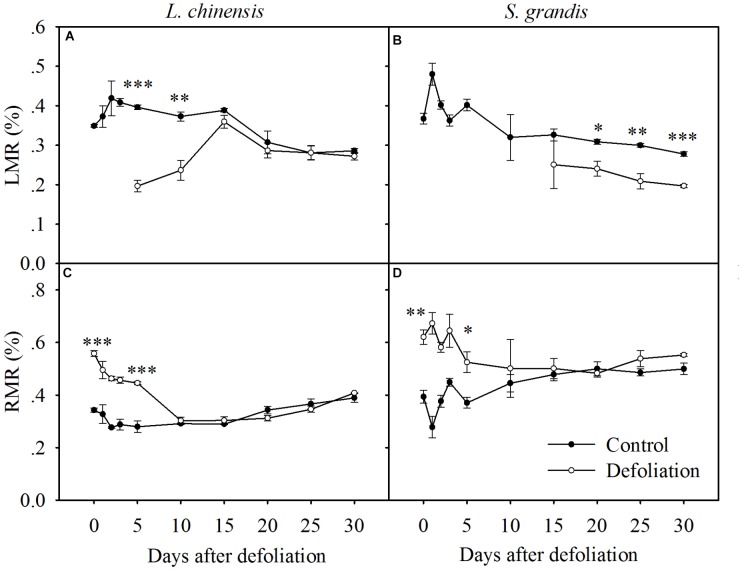
Changes in the LMR and RMR of *L. chinensis* **(A,C)** and *S. grandis* **(B,D)** after defoliation. Values are means ± SE (*n* = 3). Values and symbols have the same meanings as in Figure 1 (LMR of first 15 days for *S. grandis* is missing due to no regrowth of leaf).

The LMR of *L. chinensis* under defoliation treatment was first detected at day 5 after defoliation and then increased rapidly within the following 10 days, reaching the level of the control treatment at day 15. In contrast, the RMR of *L. chinensis* decreased within the first 10 days after defoliation and then reached the level of the control treatment. There was no significant difference in either the LMR or RMR of *L. chinensis* between the control and defoliation treatments from day 10 to day 30 or from day 15 to day 30, respectively (Figure 2 and Table 1).

The LMR of *S. grandis* under the defoliation treatment was first detected at day 5 after defoliation but was significantly lower than that of the control treatments, even after 30 days of regrowth. The RMR of *S. grandis* decreased within the first 10 days after defoliation and then reached the level of the control treatment. There was no significant difference in the RMR of *S. grandis* between the control and defoliation treatments from day 10 to day 30 (Figure 2 and Table 1).

### Defoliation Impacts on RGR

**FIGURE 3:**
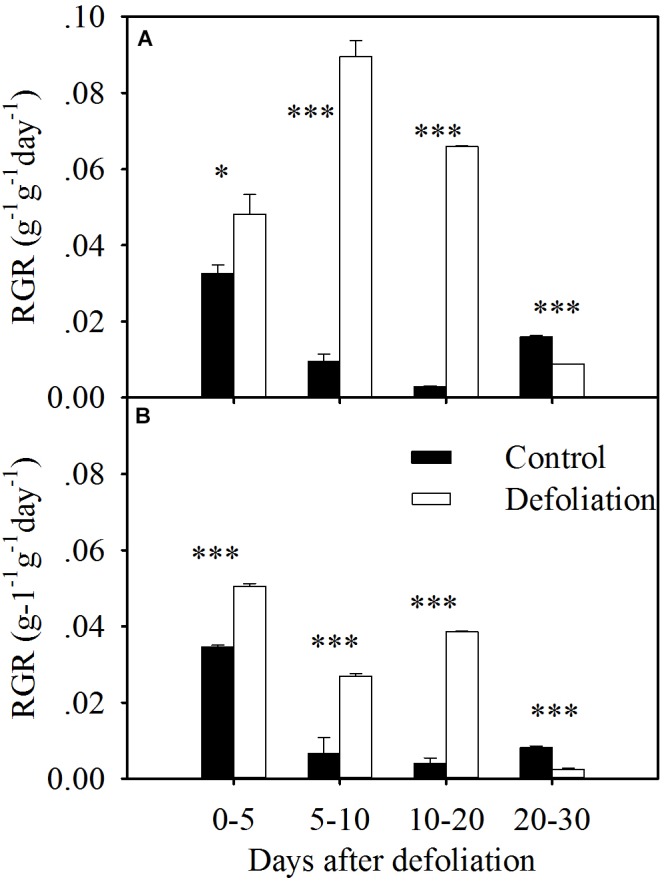
RGR in terms of aboveground biomass of *L. chinensis* **(A)** and *S. grandis* **(B)** after defoliation. Values are means ± SE (*n* = 3). Values and symbols have the same meanings as in Figure 1.

For both species, the defoliated plants exhibited a greater RGR in the first 20 days after defoliation, but the RGR of the defoliated plants within 20–30 days was less than that of the control plants (Figure 3). However, the magnitude of the increase in RGR was much greater in *L. chinensis* than that in *S. grandis* (Figures 3, 7B). On average, the RGR of *L. chinensis* under the defoliation treatment was 1.8 times that of *S. grandis*.

### Defoliation Impacts on Biomass Production

**FIGURE 4:**
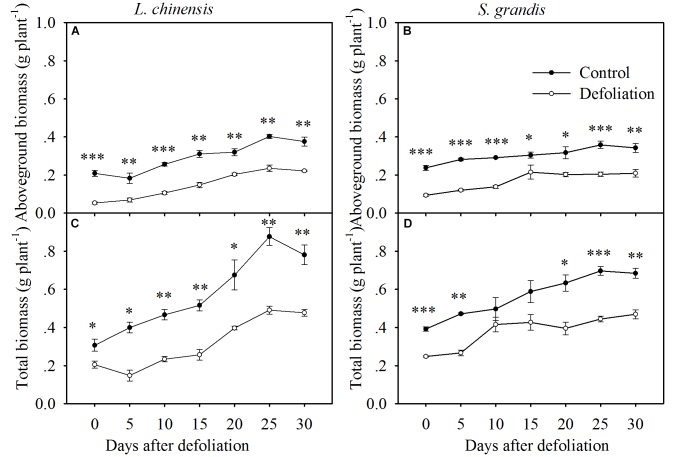
Changes in the aboveground biomass and total biomass of *L. chinensis* **(A,C)** and *S. grandis* **(B,D)** after defoliation. Values are means ± SE (*n* = 3). Values and symbols have the same meanings as in Figure 1.

**FIGURE 5:**
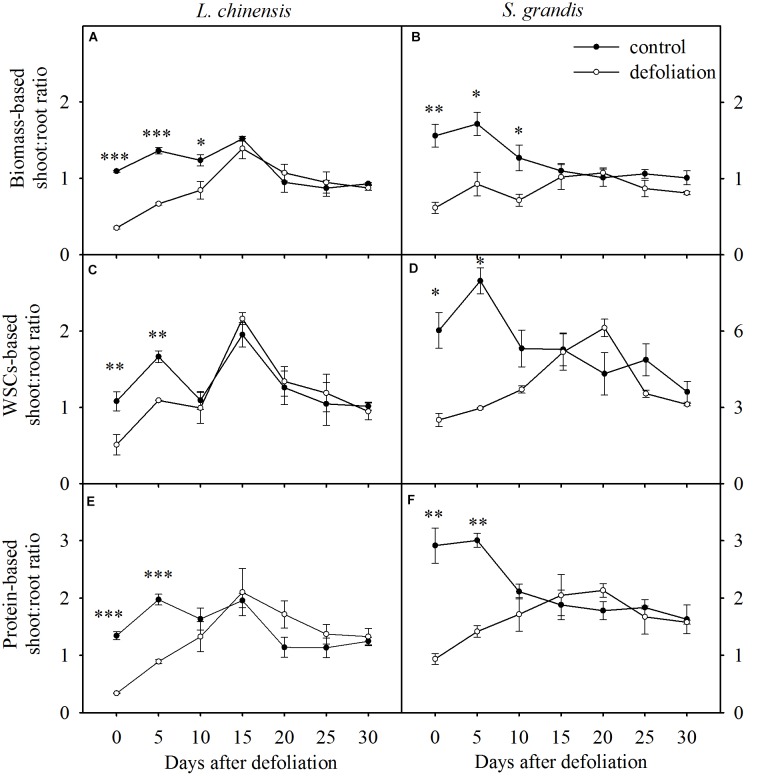
Changes in biomass-based, WSC-based, and protein-based shoot:root ratios of *L. chinensis* (**A,C,E**, respectively) and *S. grandis* (**B,D,F**, respectively) after defoliation. Values are means ± SE (*n* = 3). Values and symbols have the same meanings as in Figure 1.

Defoliation significantly reduced the aboveground biomass and total biomass of both species within 30 days of regrowth (Figures 4, 7A,B and Table 1). For *L. chinensis*, defoliation removed 74% of the aboveground biomass; as a result, after 30 days of regrowth, the aboveground biomass and total biomass of defoliated individuals were 31 and 39% less than those of the control plants. For *S. grandis*, defoliation removed 61% of the aboveground biomass, and the aboveground biomass and total biomass of defoliated individuals were 39 and 31% less than those of the control plants, respectively (Figure 5 and Table 1).

### Defoliation Impacts on Shoot:Root Ratios in Terms of Biomass, WSCs, and Proteins

The biomass-based shoot:root ratios of *L. chinensis* and *S. grandis* increased significantly within 15 days after defoliation; however, there were no significant differences between the defoliated and control plants for either species from day 15 to day 30 (Figures 5, 7D and Table 1).

The WSCs- and protein-based shoot:root ratios of *L. chinensis* and *S. grandis* exhibited a significant increase within 10 days after defoliation; however, neither the WSCs-based nor protein-based shoot:root ratio between the defoliated and control plants significantly differed for either species from day 15 to day 30 (Figures 5, 7D and Table 1).

### Biomass-Based Shoot:Root Ratio With the Amounts per Plant and Concentrations of WSCs and Proteins

**FIGURE 6:**
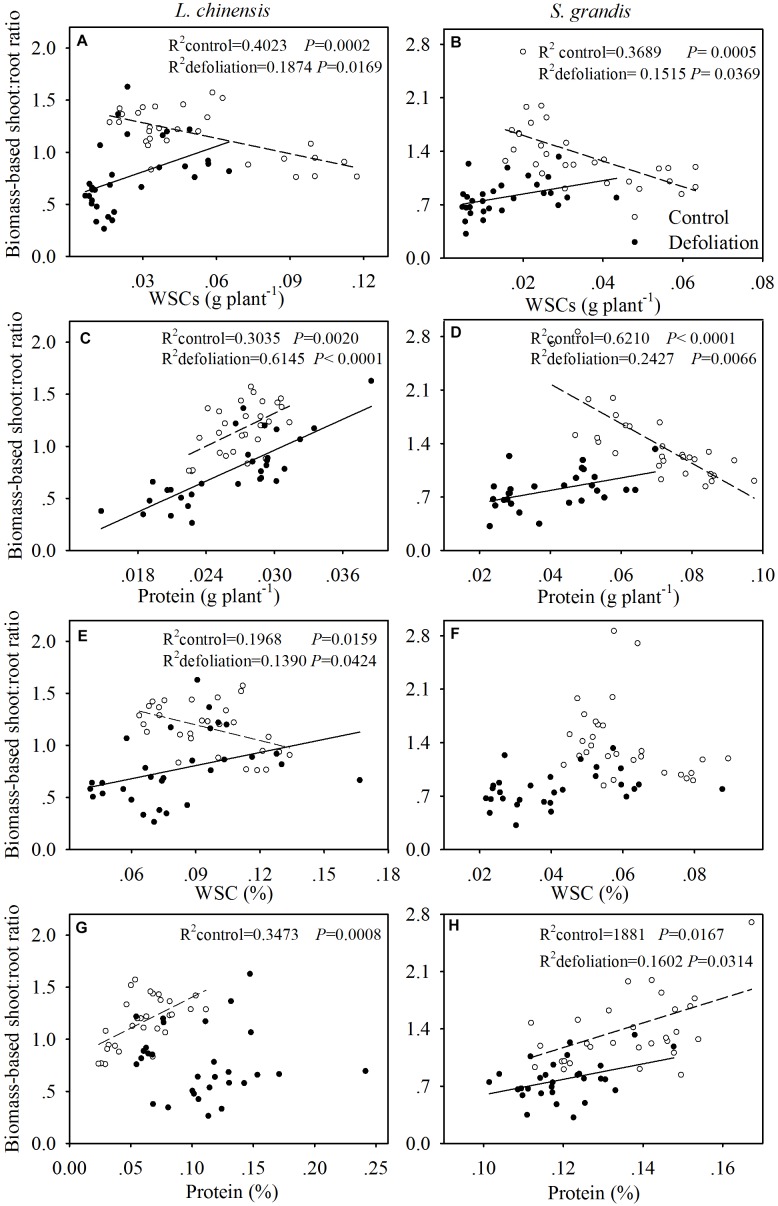
Relationships of the biomass-based shoot:root ratio with the amount of WSCs (g plant^-1^) and proteins (g plant^-1^) per plant and with the concentration of WSCs (%) and protein (%) in *L. chinensis* (**A,C,E,G**, respectively) and *S. grandis* (**B,D,F,H**, respectively) subjected to defoliation (filled circles and solid line) and control (open circles and dashed line) treatments.

Under the control treatment, the biomass-based shoot:root ratio for both species was negatively and linearly related to the amount of WSCs per plant; however, this correlation shifted to a positive linear correlation under the defoliation treatment (Figures 6A,B).

For *L. chinensis*, the biomass-based shoot:root ratio was positively and linearly related to the amount of proteins per plant under both the control and defoliation treatments (Figure 6C). For *S. grandis*, the biomass-based shoot:root ratio was negatively and linearly related to the amount of proteins per plant under the control treatment but displayed a positive and linear correlation under the defoliation treatment (Figure 6D).

For *L. chinensis*, the biomass-based shoot:root ratio was negatively and linearly related to the concentration of WSCs under the control treatment but positively and linearly related to the concentration of WSCs under the defoliation treatment (Figure 6E). No relationship was detected between the two for *S. grandis* (Figure 6F).

The biomass-based shoot:root ratio for both species generally exhibited a positive linear correlation with the protein concentration (Figures 6G,H), except for *L. chinensis* under defoliation treatment, where no relationship was detected between the two (Figure 6H).

## Discussion

Our experiment demonstrated that the two grassland species displayed both similarities and differences in their regrowth response to defoliation. Generally, both species were damaged by defoliation in plant height and biomass production. However, defoliation stimulated growth in these species, as indicated by a significant increase in the RGR in both species. Such stimulation was much stronger for *L. chinensis* than for *S. grandis*, as the RGR of *L. chinensis* under defoliation treatment was 1.8 times that of *S. grandis*. Though the response strengths in biomass production of the two species differed substantially, balance speeds of these species in terms of resource reallocation between above- and below-ground organs were the same. Specifically, both species achieved a balance of WSCs and proteins at day 10 after defoliation, while biomass was rebalanced at day 15. Moreover, such rebalance processes may be mediated by WSCs and proteins, as our analysis indicated that the biomass-based shoot:root ratio was significantly related to concentrations and the amount per plant of WSCs and protein.

### Regrowth After Defoliation

Plants alter their allocation patterns by increasing nutrient allocation to organs responsible for acquiring limited resources (Chu et al., 1992) to maximize their growth. For example, plants limited by carbon often increase their resource partitioning to the photosynthetically active leaf area. Plants suffering from defoliation first maximize their regrowth by increasing the leaf area to capture more carbon per unit of resource and invest in the photosynthetically active leaf area (Chu et al., 1992). In the present study, the LMR of *L. chinensis* increased rapidly and recovered to the control level at day 15 after defoliation, while the LMR of *S. grandis* did not recover (Figure 2). These results could be at least partially explained by the lower RGR of *S. grandis* than that of *L. chinensis* in terms of aboveground biomass.

The heights of *L. chinensis* and *S. grandis* plants, which were reduced to 3 cm with defoliation, rapidly increased during the first 10 days after defoliation (Figure 1). The rate of increase slowed over the next 20 days; as a result, the final height after 30 days of defoliation was significantly lower than that of the control treatment. This result is consistent with those of the study of Zhang et al. (2007), who found that defoliation significantly decreased plant height. The lower height of defoliated plants after 30 days of regrowth relative to that of control plants may be due to the lack of sufficient soluble nutrients, such as WSCs or protein. Previous studies have shown that the regrowth of a plant after defoliation in most cases involves replenishing WSCs and proteins from organ reserves to initiate the growth of new tillers (Donaghy and Fulkerson, 1998; Fulkerson and Donaghy, 2001).

**FIGURE 7:**
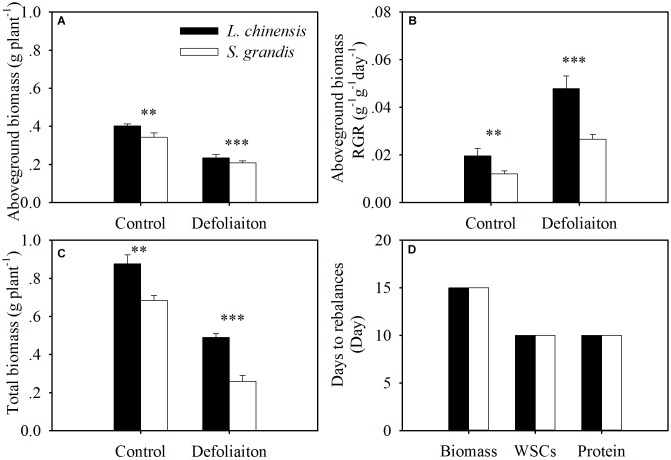
Comparison between *L. chinensis* and *S. grandis* in aboveground biomass **(A)**, aboveground biomass-based RGR **(B)**, total biomass **(C)**, and the days of restoring balance in terms of biomass, WSCs and proteins **(D)**. Values and symbols have the same meanings as in Figure 1.

The mean RGR in terms of the aboveground biomass of both species significantly increased during the 20 days after defoliation (Figures 3, 7). During the first 5 days of regrowth, translocated WSCs constituted the main nutrients (Morvan-Bertrand et al., 1999), so the mean RGR in the defoliated treatment was slightly higher than that in the control treatment. During the next 15 days, the new leaves, which contained relatively higher nitrogen concentrations, enabled plants to rapidly increase their shoot biomass; therefore, the mean RGR was much higher in the defoliation treatment than in the control treatment. As the leaf area enlarges during regrowth, plants experience a trade-off: newly assimilated carbon must be allocated to replenish reserves rather than to increase growth. After 20 days of regrowth, the defoliated plants for both species may partition more assimilates to replenish their nutrient reserves. Therefore, the RGR was lower in the defoliation treatment.

The number of tillers of both species did not significantly change after defoliation. These results are consistent with those of previous studies in that the number of tillers was not affected by defoliation (Hume, 1991; Slack et al., 2000). However, the tiller number of *L. chinensis* did increase during the 30 days in both the defoliation and control treatments, which may result from nutrient storage in rhizomes of *L. chinensis*, promoting the emergence of new tillers (Zhang et al., 2007).

### Rebalancing Processes in Biomass, WSCs, and Proteins

In this study, we observed that both species regained the necessary biomass-based rebalance approximately 15 days after defoliation. As defoliation significantly decreased the aboveground biomass and total biomass, the biomass of both plant species in the defoliation treatment did not reach that of the control treatment at 15 days after defoliation; however, the shoot:root ratio in terms of biomass did reach the level of the control. This indicated that plants function as a balanced system between above- and below-ground parts. Previous studies have shown biomass allocation between roots and shoots in response to changes in the balance between carbon and nitrogen (Reynolds and Thornley, 1982). Defoliation removes leaf tissue, which has a high nitrogen concentration and can assimilate carbon. To compensate for this loss, both species can rapidly translocate WSC reserves to support regrowth. Our extended analysis indicated that WSC concentrations in the rhizomes, roots, and stubble of *L. chinensis* decreased by 49, 44, and 41%, respectively, within the first 3 days after defoliation. For *S. grandis*, 53 and 22% of the WSCs reserved in the stubble and roots were translocated. These results highlight the role of rhizomes and stubble as the major reserve organs in perennial grasses for supplying nutrients to produce new leaves after defoliation (Hume, 1991; Donaghy and Fulkerson, 1997; Hikosaka et al., 2005).

Our results demonstrated that both species reestablished the balance in terms of WSCs and proteins between above- and below-ground organs at day 10 after defoliation, while the biomass-based rebalance was achieved at day 15 after defoliation. This suggests that the nutrient-based balances may be reached earlier than the biomass-based balance. This may be because WSCs and proteins are easier to translocate to the organs that can maximize the regrowth of plants after defoliation. Our regression analysis results show that the shoot:root ratios in terms of biomass were significantly mediated by WSC and protein concentrations and by the amount of WSCs and proteins per plant (Figure 7).

## Conclusion

This study provides us with a clear picture of the dynamic changes in regrowth and in the reallocation of resources of two grass species after defoliation in Inner Mongolia’s grasslands. For both species, defoliation impaired plant height and biomass production but increased the RGR. Both species achieved the rebalance of WSCs and proteins earlier than that of biomass, indicating that a balanced system in nutrient allocation between above- and below-ground parts is essential and important for the rebalance of biomass allocation. We deduced that the biomass-based shoot:root ratio was regulated by the concentrations of WSCs and proteins or the amounts of these nutrients per plant. As dominant species in Inner Mongolian grasslands, these two grass species cannot completely recover in terms of biomass production at day 30 after defoliation; hence, the time interval between rotational grazing activities in this area should be longer than 1 month.

## Author Contributions

YL and QP designed the research. YL, DT, and RC performed the research and analyzed the data. YL wrote the draft paper, which was revised by XY, XZ, and ZS.

## Conflict of Interest Statement

The authors declare that the research was conducted in the absence of any commercial or financial relationships that could be construed as a potential conflict of interest.
